# Hygrothermal Performance of Salt (NaCl) for Internal Surface Applications in the Building Envelope

**DOI:** 10.3390/ma15093266

**Published:** 2022-05-02

**Authors:** Vesna Pungercar, Florian Musso

**Affiliations:** Department of Architecture, School of Engineering and Design, Technical University of Munich, 80333 Munich, Germany; musso@tum.de

**Keywords:** salt, gypsum, hygrothermal performance, experiment, WUFI simulation

## Abstract

Salt (NaCl), as a by-product from the potash and desalination industry, can be the solution to the scarcity of building materials and might replace more energy-consuming materials. However, salt carries the risk of deliquescence in humid environments. This study conducted fundamental research on the hygrothermal performance of salt for internal surface applications in the building envelope in six different climate conditions. In addition, salt’s performance was also compared with that of gypsum in similar applications. The simulation models (using WUFI^®^Pro, WUFI^®^Plus) and in situ measurements were applied to investigate the hygrothermal consequences of the incorporation of salt on the thermal envelope, indoor environment, and energy consumption. Our studies revealed that salt provided the best hygrothermal responses without Heating, Ventilation, and Air Conditioning (HVAC) in very hot-dry and the worst in very hot-humid climates. With an energy-efficient thermal envelope and HVAC, salt can also find an indoor application in temperate, continental, and subpolar climates. In comparison to gypsum, salt has a slightly higher energy demand (heating, cooling, and dehumidification) due to its higher thermal conductivity and moisture resistance. This study fills the knowledge gap on salt’s hygrothermal performance and shows the potential in its utilization.

## 1. Introduction

Newly built or retrofitted buildings are expected to be energy efficient [1,2], provide comfortable indoor room conditions for living [3], and be durable [1]. While new requirements are improving buildings’ energy efficiency with higher airtightness and more insulation of the building envelope, the moisture content inside the buildings is increasing [1,4,5,6]. Too much moisture in the building envelope or too high relative humidity in the room air provide ideal conditions for mould growth [1], deterioration of the materials [7], as well as unsuitable indoor room conditions [3]. Various different strategies [8] have already been implemented in research and practice to counteract the moisture challenge, including energy-efficient building envelopes, controlled HVAC systems, improvement of occupant behaviour, and innovative building constructions [9].

In addition to these strategies, the selection of materials is an area of great interest, especially now, when the world population is growing [10] and the demand for building materials is increasing [11]. It is thus essential to identify new building materials that can substitute rare, expensive or energy-consuming materials that can contribute to better living conditions [12].

One of those potential materials is salt (NaCl), which is a by-product from the potash and desalination industries in a quantity of up to 3 billion m^3^ per year [13,14]. Typically, salt waste is discharged directly into the environment where it causes negative impacts (change in salinity, increase in temperature, and loss of biodiversity) [15,16,17,18,19,20,21,22,23,24,25,26,27,28]. However, salt can have advantages as a building material in increasing resource efficiency; it is also antibacterial [19] and inflammable [19], has no odour [20], and can store humidity and heat [19,21,22]. In terms of health, salt caves and salt rooms across Europe have been shown to positively affect human lung cancer cells, depression, respiratory, and skin-related diseases [23,24,25,26,27,28,29,30,31,32,33,34]. Salt has already been used as a building material in the past [35,36,37,38,39,40,41,42,43,44]. Initially, buildings were built from solid salt blocks cut from nearby salt-rich lakes [35,36,37]. The Romans diversified the use of salt in construction, for example, by mixing seawater, volcanic ash, and lime to create a strong concrete [38,45]. In the last 60 years, salt has entered a new round of innovation including technological developments in compressing salt under pressure [46,47,48,49], 3D printing with salt [41,42,43,50], and using natural crystallization for new products (shading system or salt plates) [51,52]. In recent years, salt blocks from the Himalayas are starting to get more attention in the construction industry due to their high salt content (up to 98.30% [44]), workability, easy fixing systems, and translucency and have already been used in several restaurants and spas worldwide [53,54]. However, in contrast with more commonplace building materials, salt must be used with caution unless in very hot-dry climates or controlled indoor conditions [44]. Limitations in the use of salt stem from the solubility of salt crystals in water and at high relative humidities (more than 75.0%) [19,22], its corrosive action on steel [55], and its detrimental and efflorescent effects on bricks [56].

Salt can be incorporated into a wide range of materials and components [39,40,41,45] and limited studies on the use of salt in the field of construction have already been undertaken [21,39,57,58]. Most of the studies have been dedicated to studying the mechanical [39,58,59] or hygrothermal [21] properties of salt mixtures such as: karshif stone (salt and clay) and salt concrete (salt and concrete). Karshif stone is a material that can be still found in Siwa Oasis in Egypt [57,60]. It was designed by collecting salt pieces from the nearby salt sea, connected by salt–clay mortar, and under very dry climate conditions over many years formed into a stone [57]. Makhlouf and his team [21] examined the hygrothermal properties of this karshif stone (a salt block composed of up to 95.0% salt and enriched with clay and sand) and compared it with sandstone and limestone. They discovered that karshif stone can buffer moisture better than sandstone and limestone. The Deutsche Gesellschaft zum Bau und Betrieb von Endlagern für Abfallstoffe mbH (DBE, Peine, Germany) has defined the mechanical and thermal material properties of salt concrete mixture (54.0% salt and 46.0% concrete) for the safe disposal of radioactive waste in Morsleben, Germany [39]. The specific heat capacity (C) and heat conductivity (λ) of salt concrete were within a range of values for concrete and salt rock. However, the porosity was higher, and permeability and compressive strength lower, in comparison with commonly used concrete. A similar research project was conducted by Czaikowski and his team [58], who investigated the chemical–hydraulic behaviour of salt concrete in contact with saturated NaCl solution. Their experimental study of sealing systems for disposal of nuclear waste in Germany resulted in more or less identical material properties as those defined by DBE.

There are very few scientific studies about salt as a building material and usually, these have focused only on salt’s material properties. Salt applications on the thermal envelope interior and hygrothermal characterization have not yet been explored. Applying salt as an interior finish to the building envelope can modify the hygrothermal performance of the exterior wall, which might result in a number of hygrothermal risks [1,3,61]. The increased water content in the wall construction and in the interior surface of salt material may exceed the critical relative humidity and water content of salt. The critical hygrothermal conditions found in the literature for salt are characterized by a water content of over 0.5% (5 kg/m^3^) at relative humidity greater than 75.0% [55,56,57]. As long as the water content (moisture) and relative humidity in the pore system of salt remain above these critical values, condensation will occur and the salt crystals will dissolve [19,22]. Additionally, salt’s higher vapour diffusion resistance factor (in comparison with gypsum) [62,63,64] might lower the temperature of the wall structure and change the drying time of the wall. Lastly, salt’s potential influence on the indoor air quality (air relative humidity and air temperature) should be investigated since it can affect the comfort and health of building occupants.

## 2. Materials and Methods

The key aims of our study are to evaluate the moisture and heat performance of salt blocks for internal surface applications in the building envelope and to investigate their influence on room temperature and humidity in different climatic regions. To achieve this, we conducted hygrothermal simulations and on-site measurements. In the hygrothermal simulation, the relevant hygrothermal properties of the salt block were firstly defined, used as input values in the simulation, and compared with gypsum. On-site measurements were typically taken for 5 months to evaluate salt behaviour in real-life situations. Our research contributes to filling the knowledge gap on the risks and benefits of using salt in the thermal envelope, which helps to understand salt’s potential as a building material and how it ages.

### 2.1. Hygrothermal Simulation

#### 2.1.1. Objective

**Figure 1:**
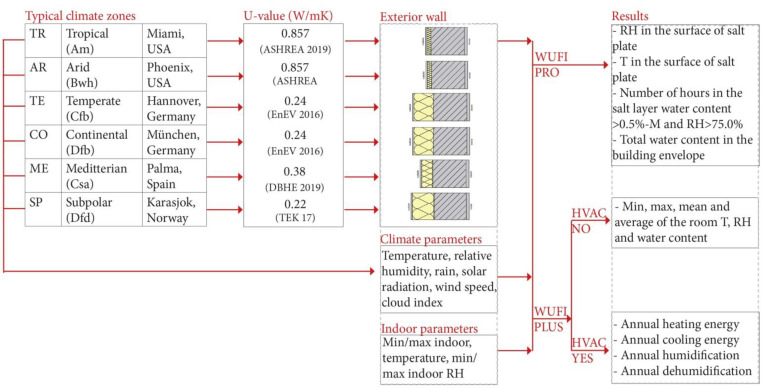
Simulation model design with the boundary conditions and expected results.

The transport of heat and moisture in the thermal envelope under natural weather conditions were simulated with WUFI^®^Pro, while the influences on the indoor air temperature and relative humidity were monitored with WUFI^®^Plus for 6 different climates. To compare the hygrothermic behaviour of the salt plate with that of a more typical interior finish, a sample with an internal gypsum plaster cladding was also studied. The WUFI^®^Pro simulation investigated the frequency of overstepping the critical boundaries and the impact on the hygrothermal process in the wall assembly/the interior surface of salt material in different climatic zones. WUFI^®^Plus simulations were carried out to define energy demand (cooling, heating, dehumidification, and humidification) and indoor air quality (see Figure 1).

#### 2.1.2. External Condition—Climate Parameters

**Figure 2:**
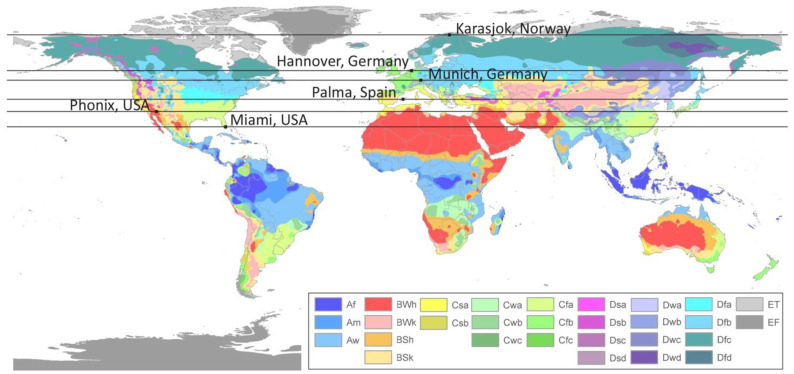
Six different climate zones in Europe and North America, according to the Köppen climate classification.

External conditions (Table 1, Figure 2) were chosen across six different climate zones in Europe and North America, according to the Köppen climate classification. These locations were selected to investigate the most appropriate climatic conditions for salt materials. Meteorological data were defined in WUFI^®^Programs and consisted of annual outdoor air temperature, annual outdoor relative humidity, mean wind speed, solar radiation sum, and rainfall sum.

#### 2.1.3. Internal Conditions—Indoor Parameters

The internal simulation conditions in WUFI^®^Pro were obtained by standard values from the WUFI database. The indoor conditions in WUFI^®^Plus varied: at first, the HVAC was turned off to evaluate the influence of the climate zone and construction on the indoor temperature and relative humidity. In the next step, the HVAC was turned on, to maintain the indoor air quality standards and to evaluate the energy demand (annual heating and cooling, humidification, and dehumidification). Table 2 lists the various hygrothermal impact indicators.

#### 2.1.4. Boundary Condition—Exterior Wall

The simulation models used a masonry construction typical in Germany with different thicknesses of external thermal insulation composite system (ETICS) (Table 1). The ETICS thickness was defined according to the locally permitted maximal heat transfer coefficient U-value of the specific climate zone (see Table 3). The simulation model (exterior wall) comprised of four main layers: (1) an outdoor render, (2) a thermal insulation, (3) a brick construction, and (4) an indoor plaster (salt or gypsum). Salt was always simulated and compared with the gypsum for a better understanding of the salt’s performance. The salt material analysed was Himalayan salt rock [53,65], which is the most common salt material in the construction industry, fixed in place with various techniques (glued on interior walls, hung on a secondary mesh construction, or connected with steel profiles) [54]. The hygrothermal properties of this salt rock could not be found in the literature and were, therefore, for the goals of this research, analysed at Fraunhofer Institut IBF, Germany in 2020 [62,63]. The relevant properties for the hygrothermal simulation of all other materials, used as input data in WUFI^®^Pro and WUFI^®^Plus, are presented in Table 3.

### 2.2. Experimental Measurements

#### 2.2.1. Objective

We took experimental measurements to investigate the hygrothermal impact of salt, gypsum, and salt–gypsum in a temperate climate. The relative humidity and the materials’ temperatures were tested over five months in Munich, Germany. The measured results were then compared with the simulation models.

#### 2.2.2. The Testing Room

**Figure 3:**
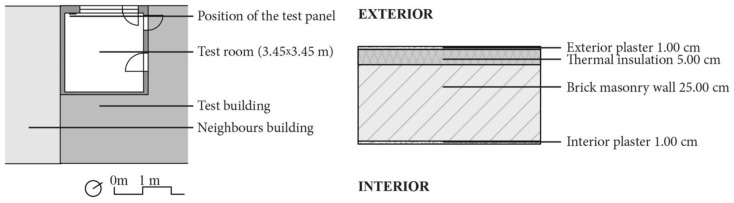
**Left**: Ground plan of the test room. **Right**: Exterior wall of the testing room.

The monitoring was carried out in a room in a typical existing 1980s residential building in Munich, Germany (48°10’ N, 11°32’ E) [9]. The room has three internal and one external walls (Figure 3). The investigated part of the room was the external wall, composed of a brick wall with poor thermal insulation, almost no wind exposure, and southwest orientation. This existing wall is made up of four layers (Figure 2) and during the day is shaded 70.0% of the time by vegetation, balconies, and surrounding buildings in the summer and 80.0% of the daytime in the winter. Four people live in the apartment, but the test room was mostly used by just two. The room’s interior conditions are not totally controlled and represent rather typical living conditions of a family with varying room occupancy, with heating in winter and shading in summer, together with influences from other rooms.

#### 2.2.3. The Test Materials

**Figure 4:**
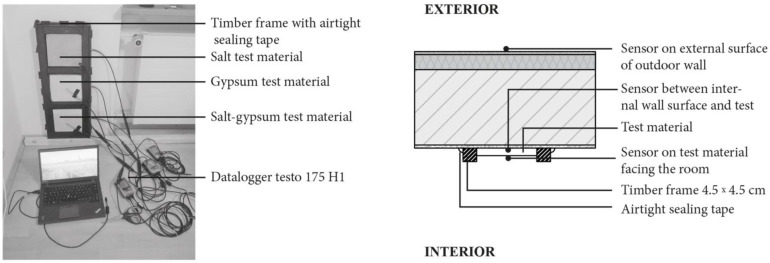
**Left**: Test panel. **Right**: Horizontal section through the test panel installation.

One test panel (Figure 4) comprised of three different materials was placed on the indoor surface of the exterior wall. It consists of a 29.0 cm × 78.0 cm timber frame filled with samples of the three materials of 20 cm × 20 cm × 2.5 cm size. From the top down, these materials are: pink rock salt, gypsum, and salt–gypsum. The material characteristics of the salt plate and gypsum are shown in Table 3. Boundary condition (construction of the exterior walls with the material properties). The salt–gypsum sample is a mixture of 70.0% gypsum and 30.0% salt: however, its material properties were not tested. The joints between the test materials and the timber frame were filled with silicone paste, while the fixing of the frame to the wall was made airtight with sealing tape. Nevertheless, we assume that the temperature difference between the internal and external surfaces of the material samples and sealing deformation cannot provide total control of moisture and temperature flow.

#### 2.2.4. The Test Instrumentation

Temperature and relative humidity from Testo were installed at the centre of each sample material surface to measure relative humidity and temperature. One sensor was installed on the centre of the outer surface facing the room, the second sensor was installed on the centre of the interface between the surface of the material and the inner surface of the external wall, and the third sensor was installed on the outer surface of the external wall (Figure 4). Indoor environment conditions were measured with temperature and relative humidity sensor 176 H1 in the middle of the room at 1.8 m height. Outdoor environment temperature and relative humidity values were taken from the real weather condition. All the sensors were calibrated by the manufacturers and the accuracy ranges are shown in Table 4.

#### 2.2.5. The Test Protocol

The duration of the monitoring was about 139 days (from 2 August 2020 till 18 December 2020), which covered the three climate conditions: hottest (summer), moderate (autumn), and coldest periods (winter). In view of the manufacturer’s recommendation for monitoring salt materials with steel sensors, their data were collected for less than 6 months. Data from the sensors on the interior wall, the test panel, and the exterior wall surface were saved every 10 min during the testing period. The interior temperature and humidity in the test room were not controlled. It changed according to its occupancy level, the heating period, the extent of shading to the window, and the infiltration of air through the doors and windows.

## 3. Results

### 3.1. Simulation WUFI^®^Pro

**Figure 5:**
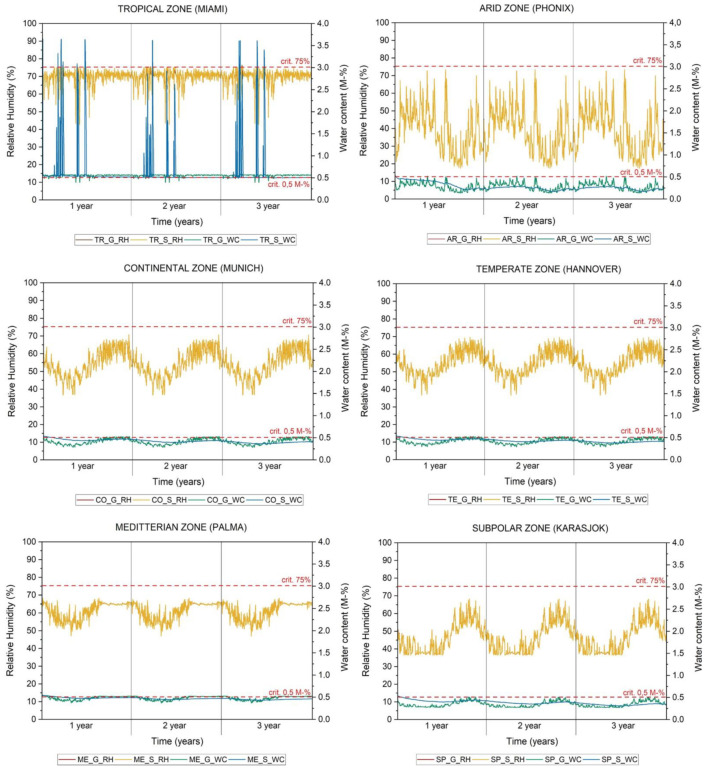
Simulated RH and water content of salt and gypsum over three years (G_RH—gypsum and relative humidity, S_RH—salt and relative humidity, G_WC—gypsum and water content, S_WC—salt and water content).

Table 5 and Figure 5 show the values of the hourly simulated relative humidity (RH), temperature (T), and water content (WC) for the indoor surface (gypsum—G and salt S) and for the exterior wall construction for each climate (TR—Tropical, AR—Arid, TE—Temperate, CO—Continental, ME—Mediterranean, and SP—Subpolar). Minimum, maximum, average, and mean values of temperature, relative humidity, and water content are listed in Table 5 to show the differences in climate zones as well as the comparison between salt and gypsum. The higher the temperature and relative humidity in a climate zone, the higher the T, RH, and WC in the observed materials.

Figure 5 shows the water content and RH over three years in the gypsum (G) and salt (S). The differences in RH in both materials are negligible compared to the water content. The water content in gypsum is more variable over time than in salt and shows a slight water uptake during the three years in all climate zones.

### 3.2. Simulation WUFI^®^Plus—Influence on the Indoor Air Quality and Energy Consumption

**Figure 6:**
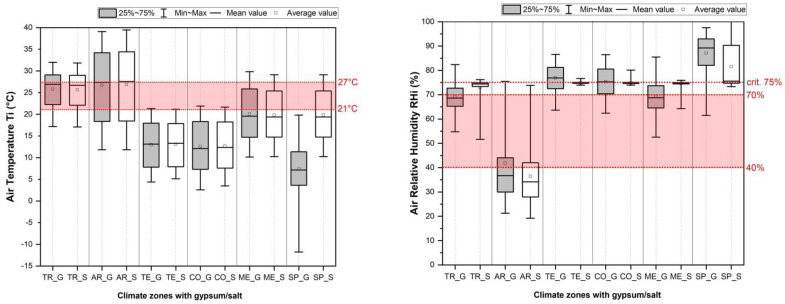
Simulated indoor air temperature and relative humidity for 6 climate zones (TR—Tropical, AR—Arid, TE—Temperate, CO—Continental, ME—Mediterranean, SP—Subpolar) with gypsum (G-grey box) or salt (S-white box) without HVAC. Red area – area for the most comfortable T (°C) and RH (%).

Figure 6 presents the dynamically simulated data of indoor air temperature and air relative humidity for gypsum and salt without HVAC (Heating, Ventilation, and Air Conditioning). The differences in the values can be attributed to the outdoor environmental influences and material parameters. The main differences (between G and S) are in relative humidity and not in temperature. The enclosed space with salt shows, in a comparison with gypsum, the lower range and, in most of the cases, a lower average RH value.

**Figure 7:**
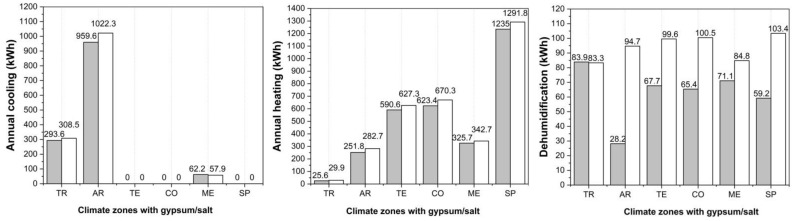
Comparison of annual cooling, heating, and dehumidification demand for gypsum and salt in six climate zones (TR—Tropical, AR—Arid, TE—Temperate, CO—Continental, ME—Mediterranean, SP—Subpolar, grey—gypsum, white—salt).

The annual energy consumption (cooling, heating, dehumidification, and humidification) with respect to outdoor environmental conditions for G and S are presented in Figure 7. These results help us understand how different surface materials influence energy consumption in various climate conditions. The result of the material influence is that in all climate zones, no energy for humidification is needed and that in most cases (13 out of 15), salt performs with a slightly higher energy demand in comparison with gypsum.

### 3.3. Measurements

**Figure 8:**
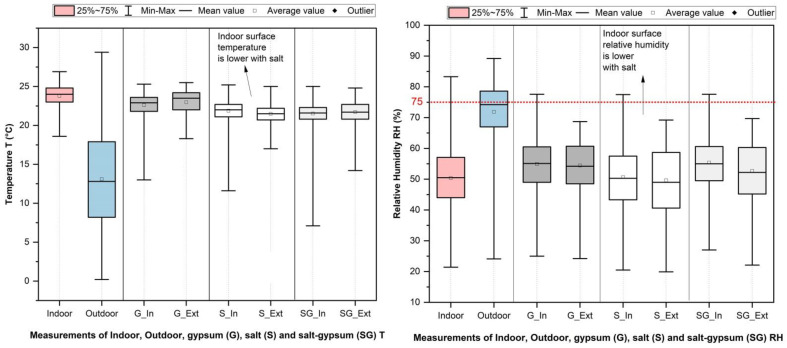
In situ measurements of relative humidity and temperature in Munich (red colour—indoor air temperature, blue colour—outdoor air temperature, grey colour—gypsum, white colour—salt, light grey colour—salt–gypsum, In—surface to interior, Ext—surface to exterior).

Figure 8 and Table 6 show the relative humidity and temperature of in situ measurements in the CO climate zone (Munich) for three materials (G—gypsum, S—salt, and SG—salt–gypsum). Each box shows the highest, lowest, mean, and average values.

## 4. Discussion

Measured and simulated data for materials are discussed with respect to three topics: temperature, relative humidity, and water content, and influence on the indoor air quality and energy consumption. For a better interpretation of the performance of salt, gypsum values are set as reference models and compared with salt.

### 4.1. Temperature

Salt (S), in comparison to gypsum (G), shows a reduction in the temperature of the surface of the internal walls. As can be seen in Table 5 and Table 6, the measured and simulated average surface temperatures of salt are, in all climate zones, slightly lower than those of gypsum. According to the simulated results for all climate zones, the average temperature decreases up to a maximum of 0.05 °C in the TR zone (from 27.73 °C to 27.68 °C). According to the measured results (S) in the CO climate zone, the indoor surface temperature decreases by 0.73 °C (from 22.3 to 21.90 °C), which is 0.01 °C higher than in the CO simulation (from 20.78 to 20.77 °C). As can be seen in Figure 8, the measured average surface temperature of the salt–gypsum (SG) of the outer surface to indoor, and of the centre of the interface between the surface of the material and the inner surface of the external wall, is between the values of salt and gypsum. In general, the T of salt (S) is found to be lower in measured and simulated results. There is a small difference in values due to different periods of examination, and indoor and outdoor boundary conditions. With the higher thermal conductivity of salt, heat in salt is more rapidly transferred (than in gypsum) and, thus, has a slightly lower surface temperature.

### 4.2. Humidity and Water Content

In the first step, relative humidity and water content in salt and gypsum are analysed, and the frequency by which the limits specified for salt are exceeded is defined (Table 5). The annual moisture balance of the whole envelope is then analysed through simulation for three years (Figure 5). In comparison to gypsum, salt shows an increase in RH. According to the simulated results for all climate zones, the average RH in the indoor surface layer of salt increases up to a maximum of 0.36% in TR (from 69.81% to 70.17%) and at the same time exceeds the RH limits. According to the measured results for salt in the CO climate zone, the RH of the outer surface of the salt test material decreases by 4.26% (from 55.01% to 50.72%). However, the simulation values show no difference. Mixing salt with gypsum (Table 6) shows also a decrease in the moisture resistance, and the measured average relative humidity of salt–gypsum increases up to a maximum of 3.04% (in comparison to salt). Only a slight difference in the relative humidity obtained for salt and gypsum was found during the three years of the simulation period (see Figure 5).

According to the simulated results for salt in all climate zones (Table 5), the average accumulated water content on the indoor surface, compared to gypsum, decreases up to a maximum of 0.12 kg/m^3^ in the ME zone (from 4.81 to 4.69 kg/m^3^) and increases up to a maximum of 0.40 kg/m^3^ in SP (from 3.43 to 3.83 kg/m^3^). Observing the results during the three-year period (see Figure 5) in different climate zones, the highest accumulated moisture content is found in salt in the TR and ME zones. The accumulated moisture content exceeds the critical limits specified for salt for 414 h in the first year, 196 h in the second year, 402 h in the third year in the TR zone, and 2 h in the first year in the ME zone. Both findings show the high risk of salt deliquescence, which is also present in the controlled indoor environment. The reason for this is the low u-value of the building envelope systems and high air humidity of this climate zone (a drying period does not occur or is too short), so the moisture remains in the material. As a general observation, it is noted that the water content values in salt in other climate zones are higher at the beginning of the period (first year) due to some initial moisture, decrease over time, and do not vary as dramatically as in gypsum, where the values fluctuate substantially with the smallest change in air RH or T.

With respect to the water content in the whole building envelope, the difference between S and G is not significant (up to a maximum of 14.0% in the SP zone). Building envelopes with salt show higher average water content in CO (5.0%), TE (7.0%), and SP (14.0%) zones and lower in TR (9.6%) and AR (6.0%) zones and the same values in the ME zone. The highest average water content (2.71 kg/m^2^ for salt) is observed in building envelopes in the hot-humid climate zone (TR) and the lowest (9.92 kg/m^2^) in the hot-dry climate. Due to the lower porosity and higher vapour diffusion resistance factor of salt (in comparison to gypsum), the high water content from construction cannot be transported as quickly towards the outdoor surface and thus dry out. In general, the smaller the insulation thickness, at a lower RH/T, the lower the relative humidity and water content of the thermal envelope. Salt is most appropriate for applications in hot-dry climates due to its lowest risk for moisture-induced damage and, therefore, higher durability of the building envelope in such climates.

### 4.3. Influence on the Indoor Air Quality without HVAC for Enclosed Spaces

The corresponding comfort range for indoor air temperature (Ti) is in the range of 21–27 °C and relative humidity (RHi) in the range of 40.0–70.0%. The simulation results (Figure 6) for a building envelope with salt with no HVAC show the average Ti as appropriate at 25 °C in the TR and 26.9 °C in the AR zones, but inappropriate at 13.09 °C in CO, 12.66 °C in TE, 19.85 °C in ME, and 19 °C in the SP zone. The average Ti with salt decreases in comparison to gypsum by up to 0.12 °C in TR (25.80 to 25.68 °C) and by 0.15 °C in ME zones (20.08 to 19.85 °C). Average Ti with salt increases in comparison to gypsum by up to 0.81 °C in AR (26.12 to 26.93 °C), 0.08 °C in CO (12.58 to 12.66 °C), 0.11 °C in TE (12.98 to 13.09 °C), and 11.27 °C in SP (7.73 to 19 °C). So, evaluating the average indoor air temperature of the enclosed space, salt shows advantages compared with gypsum in the no HVAC conditions (in four of the six climate conditions the average Ti was higher) due to the higher thermal conductivity of salt that transmits the heat quickly from outside to inside.

Figure 6 shows also the average, min, max, and mean RHi for salt and gypsum in all climate zones with no HVAC. The average RHi for salt enclosed spaces is inappropriate in all climate zones (72.93% in TR, 36.45% in AR, 74.84% in TE, 74.85% in CO, 74.54% in ME, and 82.0% in SP). All climate zones with an average RHi between 70.0 and 75.0% and a maximum >75.0% in settings without HVAC are inappropriate for salt application due to the higher risk of deliquescence. Therefore, only AR climate zones with lower RHi are suitable for salt applications. The average RHi in salt enclosed spaces has lower values of the variation in comparison to gypsum, and in four of six cases also a lower average RHi. This is due to the higher water vapour diffusion resistance factor of salt (compared to gypsum) that does not absorb so much of the indoor RH and, thus, slightly reduces the humidity buffering ability of the building envelope to regulate variations in the indoor RH levels.

### 4.4. Influence on the Energy Consumption with HVAC for Enclosed Spaces

The measured annual energy (Figure 7) use for the heating, cooling, and dehumidification presents data for the enclosed unit, both for the gypsum and salt applications. The salt enclosed space shows an annual increase in cooling demand of 4.8% in TR, 6.0% in AR, and a decrease of 6.9% in the ME zone. The annual heating demand for the salt enclosed space is higher in all zones with a maximal increase of 14.3% in the TR zone. In addition, the annual dehumidification demand for salt is higher in five of the six climate zones in comparison to gypsum. The results show that in hot-humid climate zones where there is great external heat or moisture load, the moisture and heat are transmitted through the building envelope from outdoor to indoor. In contrast, in cold climate zones with higher internal heat or moisture, the heat and moisture flow from inside to outside. The higher thermal conductivity, lower porosity, and higher bulk density of salt enable the quicker transport of heat and increase the annual demand for heating or cooling. The higher moisture resistance of salt prevents the transport of indoor humidity and increases the indoor dehumidification consumption due to interior heat gains. In future studies, it will be important to also have the real measured data for energy consumption of salt and gypsum. This would help to improve the simulation accuracy and make more accurate recommendations for salt.

### 4.5. Suggestions for Future Studies

This research is the first-ever hygrothermal study of salt (Himalayan) as a building material for indoor application in six different climate zones. The hygrothermal performance of salt showed the potential to replace gypsum, especially in hot-dry climates. However, the knowledge gained in the study about salt’s hygrothermal behaviour is limited, as it only investigated only one salt material. Therefore, the paper gives several suggestions for future studies:

- Salt mixtures with other materials for increasing resource efficiency and saving of CO_2_: The annual world production of cement is about 4.4 billion tons [66], of gypsum 150 million tons [67,68,69], and 1.1 billion tons of salt [70,71,72,73] is produced each around the world. The production of cement and gypsum is subject to substantial criticism because of its high energy demand [74,75] and the heavy impact of mining on landscapes [15,17,76,77,78]. Resources such as FGD gypsum, which currently supply approx. 50.0% of the gypsum requirement in Germany [69], is disappearing due to German energy strategies (the phasing out of coal combustion) [79,80]. By increasing the salt content in the composite material, natural resources (e.g., natural gypsum) will be protected, less energy will be needed for production and CO_2_ emissions will be reduced. Each ton of cement replaced by one ton of salt would save approximately 600 kg of CO_2_ [81] emissions.
- Hygrothermal performance of other salt composites: The simulated and on-site measurements of different salt composites (salt and concrete, salt and gypsum, salt and clay) should be analysed in detail, comparing and evaluating the passive regulation of indoor temperature and relative humidity in different climate conditions. The inclusion of other additives should also be considered for more effective heat and moisture transport.
- Durability of salt materials: Salt materials should be exposed to different humidity, temperature and different positions in the thermal envelope to investigate degradation, aging, and durability. Measured results should be compared with simulations.
- Other constructive possibilities: In this research was salt analysed only as a cladding element. Different constructive possibilities, such as supporting components in 3D printing, modular prefabricated elements, or just filling material for interior walls, should be further considered and explored.

## 5. Conclusions

This paper presents an investigation into salt’s hygrothermal performance as an indoor building component of the thermal envelope, which is compared with a reference material (gypsum) in terms of six different typical climate zones, construction types, and HVAC.

A comparison between salt and gypsum shows that salt has higher bulk density, lower porosity, lower moisture storage, higher heat transport properties, and the same heat storage capacities as gypsum. As the simulation results of the building envelope (WUFI^®^Pro) show, the salt material layer has, in comparison to gypsum: a max. 0.05 °C decrease in the material temperature and a max. 0.36% increase in material relative humidity. Another important aspect was looking at the water content of the entire building envelope in each climate zone. We found that the building envelope containing salt shows a greater average water content of up to 13.0% in cold climate zones and lower average water content in warmer climate zones. The same influence of cold or warm climate zones on water content in the thermal envelope can reasonably be supported by the studies of Qin et al. [82] Liu et al. [83], Corrado et al. [84] and Qin et al. [85]. The highest risk of salt deliquescence is observed in TR and ME climate zones, with the lowest risk in the AR zone.

Due to the limitation of the in situ measurements, only the T and RH near the indoor and outdoor surfaces of the tested materials are measured. The simulated results show good agreement with in situ measurements for salt and gypsum in the CO zone. Both results show the same tendency in values of material temperature and humidity. However, the measured temperature at the indoor surface is slightly higher and the RH slightly lower than the simulated ones, probably due to the tested materials being located near the central heating element from 26 September 2019 till 18 December 2019. Previous studies by Moujalled et al. [86] and Illomets et al. [87] have also found that in situ measurements show slightly different results as simulations due to the heating system. However, the modelling method is shown to be correct, but will have to be adapted to real living conditions such as building envelope, occupation (behaviour and density), and energy system in the future.

The annual simulation for energy consumption in WUFI^®^Plus shows that salt has a slightly increased heat and decreased moisture transport, which leads to more cooling, heating, and dehumidification energy. However, salt has advantages in increased heat transport that reduce the indoor surface temperature, the peak of indoor air temperature, and is moreover beneficial for better indoor comfort in very hot climate zones. Decreased moisture transport in salt shows it can help to reduce the influence of the external environment RH with respect to indoor RH or it can prevent the condensed water and indoor moisture from drying out. This result correlates with other works in which the hygrothermal performance of materials in the building envelope have been studied [1,3,61,87].

The outcomes mentioned above can form the basis for some recommendations on the application of salt in internal spaces. In general, for buildings without HVAC only very dry and hot outdoor climatic environments (AR zone) are suitable as there is no risk of salt deliquescence, while the salt has a positive influence on the Ti/RHi and durability of the thermal envelope. For buildings with good thermal envelopes and controlled HVAC, the CO, TE, and SP zones might also come into consideration.

## Figures and Tables

**Table 1:** Exterior weather conditions.

	Climate Zones—Köpper–Geiger Climate Classification	City	Climate	Position (Latitude, Longitude)	U-Value Requirements (W/m^2^K)
TR	Tropical (Am)	Miami, USA	Monsoon	25.80° N 80.27° W	0.857 (ASHREA 2019)
AR	Arid (Bwh)	Phoenix, USA	Dessert, hot arid	33.43° N 112.02° W	0.857 (ASHREA 2019)
TE	Temperate (Cfb)	Hannover, Germany	Humid and warm summer	52.37° N 9.37° E	0.24 (EnEV 2016)
CO	Continental (Dfb)	Munich, Germany	Fully humid, cool summer	48.13° N 11.72° E	0.24 (EnEV 2016)
ME	Meditterian (Csa)	Palma, Spain	Dry summer, hot summer	39.56° N 2.65° E	0.38 (DBHE 2019)
SP	Subpolar (Dfd)	Karasjok, Norway	Fully humid cold summer	69.47° N 25.49° E	0.22 (TEK 17)

**Table 2:** Outdoor and indoor conditions for the simulation model.

	WUFI^®^Pro	WUFI^®^Plus
Outdoor condition (weather data)	Real weather data from the WUFI^®^Pro/Plus programme	Real weather data from the WUFI^®^Pro/Plus programme
Indoor condition	USA: ASHRAE 160 Europe: EN 15026, DIN 4108, WTA 6-2	USA: ASHRAE 160 Europe: EN 15026, DIN 4108, WTA 6-2
Component (wall/room)	Thermal envelope	Room (3 m × 3 m × 3 m)
Calculation Period, Profiles	3 Years (time steps: 1 h)	1 year (time steps: 1 h)
Orientation	Wall component is oriented to north (the lowest solar radiation)	No windows to evaluate the influence of the climate zones and construction
Inclination	90°	90°
Initial moisture and temperature in construction component	RH = 70.0% T = 20 °C	RH = 70.0% T = 20 °C
Driving Rain Coefficients	0.07	0.07
Monitor Position	Material surface	In a room
Number of occupants	1 person per room	1 person per room
Office indoor heat and moisture load	Standard program input	Convective heat: 33.3 W Radiant heat: 25.2 W, Moisture 17.55 g/h, CO_2_: 20.79 g/h Human activity: 1.2 met Air velocity: 0.1 m/s
Clothing	Standard program input	0.7 clo
Occupancy Period	Standard program input	7.00–18.00
Energy system	Only heating Depending on the climate zone (norms: EN 15026, DIN 4108, WTA 6-2, ASHRAE 160)	HVAC on: Indoor air temperature 21–27 °C RH 40.0–70.0% Max CO_2_: 3000 ppmv Air exchange: 0.6 h^−1^ Heating, cooling, humidification, and dehumidification calculated HVAC off

**Table 3:** Boundary condition (construction of the exterior walls with the material properties). The indoor material layer is gypsum or salt (dark grey).

Construction from Outside to Inside (cm)							
		U-Value (W/m^2^K)	Mineral Plaster	Mineral Insulation Board	Solid Brick Masonry	Gypsum Plaster	Salt
Wall 1: Tropical (TR) and arid (AR) climate zone	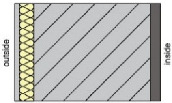	0.72 (gypsum)	1.0	3.0	24.0	2.0	
		0.77 (salt)	1.0	3.0	24.0		2.0
Wall 2: Temperate (TE) and continental (CO) climate zone	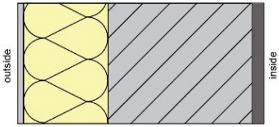	0.23 (gypsum)	1.0	14.0	24.0	2.0	
		0.24 (salt)	1.0	14.0	24.0		2.0
Wall 3: Mediterranean (ME) climate zone	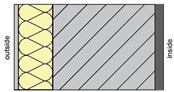	0.35 (gypsum)	1.0	9.0	24.0	2.0	
		0.35 (salt)	1.0	9.0	24.0		2.0
Wall 4: subpolar (SP) climate zone	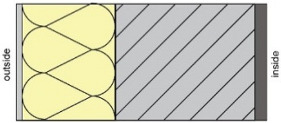	0.21 (gypsum)	1.0	16.0	24.0	2.0	
		0.22 (salt)	1.0	16.0	24.0		2.0
**Material properties**							
Bulk density (kg/m^3^)			1900	15	1900	850	2087
Porosity (m^3^/m^3^)			0.24	0.95	0.24	0.65	0.04
Specific Heat Capacity (J/kgK)			850	1500	850	850	850
Water Vapour Diffusion Resistance Factor (−)			25	30	10	8.3	7836
Thermal conductivity (W/mK)			0.8	0.04	0.6	0.2	2.65
Typical Build-In Moisture (kg/m^3^)			210	44.8	100	400	999

**Table 4:** Sensors.

Sensors	Accuracy Ranges
testo 176 H1—Temperature and humidity data logger (data logger for sensors on the test materials)	±0.2 °C (−20 to +70 °C) ±1 Digit ±0.4 °C (Remaining Range) ±1 Digit dependent on probe selected (0.0 to 100.0% RH)
Thin humidity/temperature probe with cable (sensors on the test materials)	±0.2 °C at 0 to +40 °C ±2.0% RH at +25 °C (2.0 to +98.0% RH) ±0.08% RH/K (k = 1), long-term stability: ±1.0% RH/year
testo 175 H1—Temperature and humidity data logger (exterior and interior measurements)	±0.4 °C (−20 to +55 °C) ±1 Digit at −20 to +55 °C ±2.0% RH (2.0 to 98.0%) at +25 °C ±0.03% RH/K ±1 Digit <±1.0% RH/year drift at +25 °C

**Table 5:** The characteristics of the simulated relative humidity, temperature, and water content in gypsum/salt and the water content in the whole exterior wall in different climate conditions: the minimum, the maximum, the average, and the mean range.

	TR_G	TR_S	AR_G	AR_S	CO_G	CO_S	TE_G	TE_S	ME_G	ME_S	SP_G	SP_S
**Relative Humidity, Surface (%)**												
Min	43.85	42.68	17.58	17.38	36.95	36.60	36.94	36.62	47.19	46.95	36.61	36.31
Max	89.02	99.61	71.53	73.50	68.64	70.77	68.11	69.65	74.53	76.11	66.68	68.30
Ave	69.81	70.17	38.92	39.02	54.27	54.27	54.80	54.84	61.04	61.08	46.37	46.42
Mean	70.76	70.92	38.80	38.83	53.80	53.74	54.34	54.32	62.73	62.91	45.81	45.77
**Water Content, Surface (kg/m^3^)**												
Min	3.95	4.95	1.33	1.66	2.82	3.64	2.91	3.79	3.69	4.29	2.72	3.07
Max	6.83	36.52	5.23	5.40	5.32	5.41	5.40	5.42	5.69	41.01	5.19	5.38
Ave	5.57	6.47	2.84	2.77	4.14	4.25	4.18	4.33	4.81	4.69	3.45	3.83
Mean	5.63	5.12	2.85	2.59	4.10	4.25	4.13	4.31	4.97	4.68	3.34	3.81
**Salt (Water Content > 0.5 kg/m^3^ and RH > 75.0%), Gypsum (T = 5–40 °C and RH > 80.0%) (1st year, 2nd year, 3rd year)**												
Hours	19.0, 0.0, 0.0	414.0, 196.0, 402.0	0.0, 0.0, 0.0	0.0, 0.0, 0.0	0.0, 0.0, 0.0	0.0, 0.0, 0.0	0.0, 0.0, 0.0	0.0, 0.0, 0.0	0.0, 0.0, 0.0	2.0, 0.0, 0.0	0.0, 0.0, 0.0	0.0, 0.0, 0.0
**Temperature, Surface Layer (°C)**												
Min	20.51	19.95	19.71	19.56	19.05	19.03	19.18	19.16	19.24	19.20	18.54	18.50
Max	32.43	32.40	41.47	41.43	24.88	24.88	24.82	24.83	25.20	25.23	23.99	23.95
Ave	27.73	27.68	28.20	28.17	20.78	20.77	20.77	20.75	22.56	22.55	19.66	19.65
Mean	27.86	27.79	28.14	28.13	19.65	19.64	19.70	19.69	22.88	22.85	19.43	19.42
**Water content, whole construction (kg/m^2^)**												
Min	2.81	2.36	0.39	0.38	1.68	1.51	1.78	1.59	2.15	1.95	1.65	1.34
Max	3.58	3.83	3.17	3.16	3.47	3.63	3.33	3.49	3.52	3.95	3.30	3.56
Ave	3.10	2.71	0.98	0.92	2.20	2.32	2.26	2.43	2.53	2.53	2.08	2.42
Mean	3.11	2.65	0.98	0.73	2.16	2.19	2.23	2.31	2.47	2.46	1.97	2.51

**Table 6:** In-Situ measurements of relative humidity (RH) and temperature (T) on the interior (In) and exterior (Ext) surface of three materials (S—salt, G—gypsum, SG—salt–gypsum).

	S_In RH	S_In T	S_Ext RH	S_Ext T	G_In RH	G_In T	G_Ext RH	G_Ext T	SG_In RH	SG_In T	SG_Ext RH	SG_In T
Min	33.46	18.90	33.31	18.40	39.93	18.91	41.24	18.57	41.97	18.84	3817	18.57
Max	70.86	24.99	68.11	24.76	72.72	25.06	68.25	24.91	73.21	24.82	69.00	24.65
Ave	50.72	21.90	49.70	21.52	55.01	22.63	54.52	23.00	55.42	21.54	52.74	21.74
Mean	50.01	21.88	49.43	21.46	54.27	22.88	53.46	23.52	54.21	21.45	52.71	21.69
